# Supplementary material for: Learning curve and surgical outcome of robotic assisted colorectal surgery with ERAS program
Source: Sci Rep. 2022 Nov 29;12:20566. doi: 10.1038/s41598-022-24665-w (PMC9709162; doi:10.1038/s41598-022-24665-w)
Supplement: Supplementary file 2 — Supplementary Information 2. [file 41598_2022_24665_MOESM2_ESM.zip › RAL Raw data-Ñ[▒K/056/HS_ñw╝╨░O▒Kñσ.pdf]

正式摘要(電子簽章：000000209200871)		
W55 / W55 ☐ 英文標題(注意:無英文姓名-請補病人基本檔!)	☐ 【讀取】憑證	

臺中榮民總醫院
出院病歷摘要

轉入號 : 轉出號 :

[redacted]

**(8)**流水編號	
**(9)**入院日期	110/01/24 (2021) 直腸外科 W55 - 121
**(10)**轉科日期	
**(11)**出院日期	110/01/29 (2021) 住院天數計 5 日

診斷

- 入院 :Rectal cancer
  hypertension
- 出院 :rectal cancer s/p robot assisted LAR on 2021/1/25
  hypertension

主訴

- small caliber of stool appearance for more than two month

病史

- This is a 69 y/o female with the past history of
  1. Leukocytoclastic vasculitis, s/p Plaquenil and Colchicine
  2. HTN, under norvasc
  This time, she came to our hospital due to small caliber of stool appearance for more than two month. The patient deveolped bowel habit chagne since the beginning of 2020 with mild blood-tinged stool off and on. She received fecal occult blood test biannually and all showed negative result. She recieved sedative colonoscopy no 12/16 and showed one 3-4cm in size polyp up to cecum at 15cm FAV and biopsy report showed adenoma with high grade dysplasia. CT abdomen on 12/17 showed focal asymmetrical wall thickening over rectum, malignancy couldn't be ruled out. ESD was done on 2020/12/30 and biopsy showed adenocarcinoma. Thus, she was admitted for surgical intervention.

體檢發現

-
  20201216: sedative colonoscopy: up to cecum, normal colonic mucosa, one 3-4cm in size polyp at15cm FAV, s/p biopsy and hemoclipping localization. Hemorrhoid(+). suggest ESD.
  - General appearance :
    - easy looking , conscious clear , without cardiopulmonary distress
    - Vital signs自動帶入date 體溫(BT) 脈搏(P) 呼吸(R) 血壓(BP) 身高(BH) 體重(BW) BMI
      20210124 36C 71/min 16/min 161/71mmHg 153.9cm 49.4Kg 20.9
  - HEENT :
    - Eyes: no anemic conjunctiva, no icteric sclera, normal EOM
    - ENT: no otorrhea, no rhinorrhea, no erythematous mucosa, no injected tonsil, no deformity
  - Neck: supple, freely movable, trachea midline,no deformity, no engorged jugular vein , no palpable thyroid or lymph node enlargement
  - Chest:
    - Inspection: Grossly normal, symmetric and fully expansion, no accessory muscle used, no operation scar, no spider angiomata, no gynecomastia
    - Palpation: no palpable mass or nodule, no subcutaneous emphysema, no tenderness
    - Ausculation: clear breathing sound
  - Heart:
    - Percussion: normal heart size
    - Ausculation: regular heart beat, normal S1, S2, no gallop, no murmur

-
  - Abdomen:
    - Inspection: in
      - flat contour, symetrically, no scar, no stria, no dilated veins, no rashes
    - Ausculation: normoactive bowel sound, no abdominal bruits
    - palpation: soft, no muscle guarding or rigidity, no local tenderness, no rebounding pain, no palpable mass, no palpable pulsation
    - Percussion: no shifting dulllness, no hepatomegaly, no splenomagaly
  -

Peripheral pulsation :	CA	BA	RA	FA	PA	PTA	DPA
Right side :	++	++	++	++	++	++	++/+++
Left side :	++	++	++	++	++	++	++/+++

  - Genito-anus: no tenderness or palpable mass lesion or rectal shelf, normal external genitalia, normal anal tone, brown stool

CRS CONDITION

-
  - Lung

手術日期及方法

- 手術日期及方法20210125 1. Robotic (XI) assisted anterior resection
  2. Intraoperative colonoscopy : (-).
  3. High ligation (+) : At IMA root. IMV were also ligated. # Left gonadal vein was also ligated.
  4. Take down from the splenic flexure : (-)
  5. Robotic endoGIA (45-3.8) X2 + EEA (29mm) were applied
  6. Proximal cut end: 8 cm, distal cut end: 2 cm
  7. ADHESION BARRIER FILM was placed below the central abdominal wound

住院治療經過

- After admission, operation with robot assisted LAR was done on 2021/1/25. We let her try water and then diet under ERAS protocol. She had vomiting twice on POD1 so we adjusted PCA and gave Kytron. Under the stable condition, she was discharged on 1/29.

合併症

- nil

一般檢查

- 累積報告 :

DATE	TIME	ALB	TP	BIL.T	BIL.D	ALKP	AST	ALT	LDH	CHOL	TG	HDL-C	LDL	UA	NH3	AMY
1100124	1848	4.4	6.9	0.85	0.19	116	33	23	214							
1100125	2016	3.7		0.9			30	21								

DATE	TIME	WBC	RBC	HGB	HCT	MCV	PLT	BLST	NEUT	BAND	SEG	LYM	MON	EOS	BAS
1100124	1821	4150	4.46	13.8	42.0	94.2	230		51.8			40.5	5.5	1.2	1.0
1100125	2016	8260		13.2	39.7		211		93.8			5.0	1.1	0.0	0.1

DATE	TIME	NA	K	CL	CA	BUN	CREAT	Mg	P	CRP	ESR	PCT	LIPASE	LACTATE
1100124	1848	142	4.0	105	9.2	16	1.04							
1100125	2016	141	3.7		8.1	7	0.91							

DATE	TIME	GLUCOSE	PAB	TRF	ZN	CU	KETONE	CK	CKMB	PT-P	PT-C	APTT-P	APTT-C	TRO-T
1100124	1823									10.5	10.3	27.0		

特殊檢查

病理報告

其它

出院時情況

- 3.改門診治療

出院指示

-

藥名	劑量	劑量單位	用法	頻次	數量	數量單位	現況	天數	單位
Ultracet tab	1	TAB	PO	Q8HPRN	9	TAB	TH	3	天
$Biofermin tab	2	TAB	PO	TID	30	TAB	TH	5	天
C.B. oint 5gm		TUB	TOP	TID	0	TUB	TH	1	瓶
MOSAD tab 5mg	1	TAB	PO	TID	15	TAB	TH	5	天
$Lederscon tab	1	TAB	PO	QID	20	TAB	TH	5	天

出院計劃

主治醫師 2062F 蔣鋒帆 / 住院醫師 2331F 陳凱祥

正式摘要(**電子簽章：000000209200871)**

W55 / W55

doctorRank:B
